# Assessing the clinical relevance of MRI findings in adult achondroplasia patients with lumbar spinal stenosis

**DOI:** 10.1016/j.bas.2025.104374

**Published:** 2025-07-28

**Authors:** Husule Cai, Chady Omara, Carmen L.A. Vleggeert-Lankamp

**Affiliations:** a Department of Neurosurgery, Leiden University Medical Centre, Leiden, the Netherlands; b Computational Neuroscience Outcome Center, Brigham and Woman's Hospital, Harvard Medical School, Boston, USA; c Spaarne Gasthuis Haarlem/Hoofddorp, the Netherlands

**Keywords:** Achondroplasia, Lumbar spinal stenosis, Schizas scales, Dural sac cross-sectional area, Lumbar decompression, Neurologic claudication

## Abstract

**Introduction:**

Achondroplasia (Ach) is the most common form of dwarfism, and lumbar spinal stenosis (LSS) becomes the primary problem for adult Ach patients.

**Research question:**

This study aims to determine the cutoff values of LSS for surgical decompression.

**Material methods:**

MRIs of adult achondroplasts with symptomatic lumbar spinal stenosis referred to our Medical Centre between 2019 and 2022 were reviewed. The degree of lumbar spinal stenosis was assessed by the Schizas scale and dural sac cross-sectional area (DSCA). Regression analysis was used to evaluate the association between treatment decisions and the degree of stenosis, while receiver operating characteristic (ROC) analysis was performed to identify cutoff values. A follow-up survey was conducted to validate clinical effectiveness.

**Results:**

In total, 68 patients were included (mean age: 46.2 ± 15.4 years; 60 % female). Individuals subjected to decompression had higher Schizas scales (p < 0.001) and smaller DSCA (62.5 ± 34.3 mm^2^ vs 91.0 ± 40.2 mm^2^, p < 0.001). ROC analysis demonstrated that lumbar decompression was indicated at levels with Schizas grade C or D or a DSCA less than 62 mm^2^. Follow-up investigation revealed less favorable clinical outcomes in patients exceeding the severity thresholds of either the Schizas scale or DSCA.

**Discussion and conclusion:**

Schizas scales C and D or DSCA smaller than 62 mm^2^ should alert the surgeons that the non-surgical approach is prone to fail. Furthermore, this threshold also facilitate clinically relevant interpretation of lumbar MRIs by non-specialists in achondroplasia patients.

## Highlights

• In achondroplasia patients, upper lumbar spine is often affected; L_23_ is the most stenotic level.
• Schizas scales C/D or DSCA < 62 mm^2^ in lumbar stenosis correlate to neurogenic claudication requiring surgery.
• Patients with Schizas grade C/D or DSCA <62 mm^2^ tend to have worse outcomes: higher ODI and lower mJOA scores.

## 1 Introduction

Achondroplasia (Ach) is the most common form of dwarfism. The disorder is caused by a gain-of-function mutation in Fibroblast Growth Factor Receptor 3, which is inherited as an autosomal-dominant trait (Rousseau et al., 1994; Shiang et al., 1994). The incidence of achondroplasia is estimated to be approximately one in every 15,000 to 40,000 live births (Aryanpur et al., 1990; Morgan and Young, 1980). Characteristic clinical manifestations of achondroplasia include small stature, rhizomelic shortening of limbs, macrocephaly, thoracolumbar kyphosis, and stenosis of the spinal canal (Pauli, 2019).

Lumbar spinal stenosis (LSS) is a common problem of adult achondroplasts (Pauli, 2019; Unger et al., 2017). LSS symptoms constitute clinical manifestations ranging from low back pain and radiating pain into the buttocks or the legs, exacerbated by sustained walking, standing, or lumbar extension (Schkrohowsky et al., 2007; Kreiner et al., 2013). Bowel and bladder incontinence or paraplegia could occur with the progression of severity (Ornitz et al., 2017). Approximately, 10 %–30 % of adult Ach patients suffer from lumbar spinal stenosis symptoms (Fredwall et al., 2020; Hunter et al., 1998), and it has been described that 23 % of adult individuals are subjected to lumbar decompressive surgery during their lifetime (Mahomed et al., 1998).

Surgical decision-making for lumbar spinal stenosis requires a comprehensive evaluation of both clinical symptoms and radiological findings. However, in patients with achondroplasia, the atypical spinal canal morphology resulting from their altered lumbar anatomy poses a challenge for accurate radiological assessment, especially for non-specialists. This study aims to establish radiological thresholds to guide lumbar decompression surgery. The clinical utility of these cutoff values will be further validated by postoperative outcomes obtained through longitudinal follow-up.

This study employed the scoring of the Schizas scale and measured the dural sac cross-sectional area (DSCA) to quantify stenosis of the lumbar spinal central canal. Schizas scales assess the lumbar spinal stenosis from the perspective of the morphological relationship between cerebrospinal fluid and nerve rootlets (Schizas et al., 2010).

By evaluating MRI findings in conjunction with treatment decisions, we aim to present cutoff values to identify lumbar levels indicated for surgical treatment. Furthermore, clinical outcome data will confirm the effectiveness of the proposed thresholds. The findings could assist non-specialists in interpreting lumbar MRI scans with greater clinical relevance.

## 2 Material and methods

### 2.1 Data source

Radiological and clinical data of achondroplasia patients visiting our outpatient clinic between Jan 2019 and Dec 2022 with complaints of neurogenic claudication were reviewed. Diagnosis and clinical symptoms were confirmed by a senior neurosurgeon dedicated to spinal surgery in achondroplasia. Individuals who had lumbar spinal surgery before visiting the outpatient clinic were excluded from this study, and those without lumbar MRI scans were also excluded. The ethical statement has been approved by the medical ethical committee Leiden Den Haag Delft (NL43032.058.13) and informed consent was also obtained from all the enrolled patients.

### 2.2 MRI assessment

Sagittal and axial plane T2-weighted MRI scans were evaluated. The grade of spinal stenosis was evaluated at the disc levels, where stenosis is usually most prominent between T_12_L_1_ and L_5_S_1_ levels. Some MRIs did not comprise an axial view of all lumbar levels, and those levels were omitted from the assessment.

For patients maintaining conservative therapy, the earliest available MRI was selected for analysis. For those undergoing surgery, MRI images closest to (before) surgery were selected.

The Schizas scale is a 7-grade system, with A1-A4 detailing mild stenosis. As mild stenosis is not an indication for surgery, this study adapted the Schizas scales into a 4-grade system by merging A1 to A4 into ‘A’. Schizas scales represent moderate stenosis as grade B, severe stenosis as grade C, and complete obliteration of the canal as grade D (Schizas et al., 2010).

**Fig. 1:**
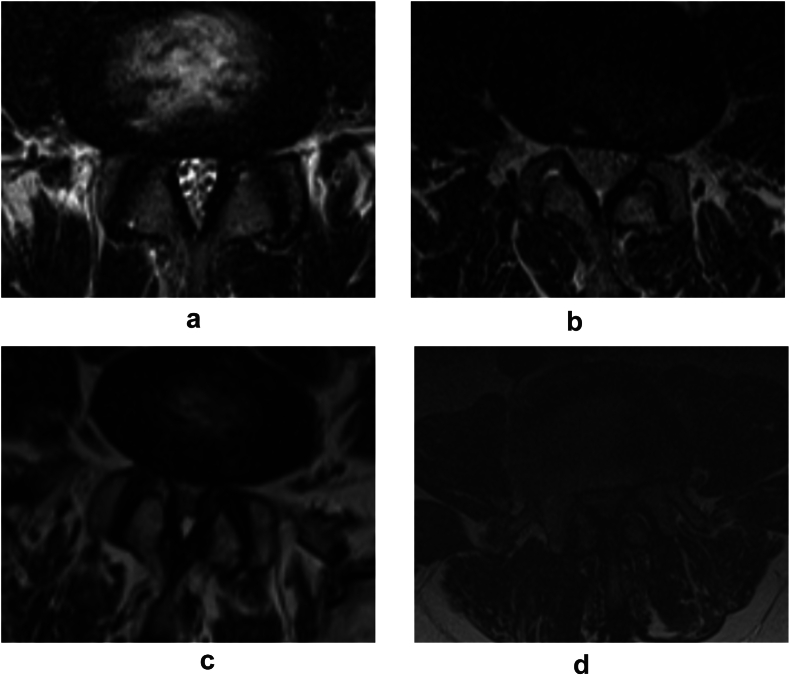
Schizas scales assessments performed on axial spinal T2-weighted MRI. Schizas grade A; The CSF is visible due to the slight stenosis, with congestion at the nerve root (Fig. 1a). Schizas grade B; The entire dural sac is occupied by the nerve roots, giving the CSF a “speckled” appearance with neural roots scattered throughout (Fig. 1b). Schizas grade C; Epidural fat is still present, but no rootlets are discernible (Fig. 1c). Schizas grade D; A uniform gray signal is seen, with no discernible nerve roots and epidural fat (Fig. 1d).

The assessment is as follows:

Schizas grade A (Normal & Mild stenosis): The dural sac's CSF is readily visible due to the slight stenosis. Although it is distinct, there may be congestion at the nerve root (Fig. 1a).

Schizas grade B (Moderate stenosis): The entire dural sac is occupied by the nerve roots. The nerve roots become crowded, giving the CSF a “speckled” appearance with neural roots scattered throughout (Fig. 1b).

Schizas grade C (Severe stenosis): Epidural fat is still present, but no rootlets are discernible, resulting in total effacement of CSF (Fig. 1c).

Schizas grade D (Complete obliteration): A uniform gray signal is seen, with no discernible nerve roots or epidural fat (Fig. 1d).

The MRIs were screened and graded by two independent researchers (HC and OC) and a kappa interobserver rate was calculated. In case of discrepancy, agreement was reached in a consensus meeting, in which the senior neurosurgeon dedicated to achondroplasia spinal surgery (CVL) had a final vote.

**Fig. 2:**
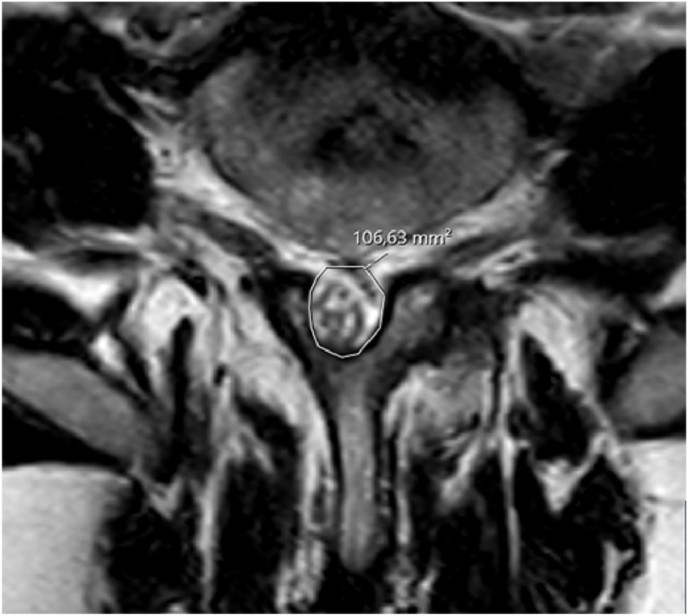
Axial T2-weighted MRI scan demonstrating the dural sac cross-section areal (DSCA). The white line indicates the outline of the DSCA, which is 106.63 mm^2^.

The DSCA was measured at the corresponding MRI images at which the Schizas scales were assessed. DSCA was defined as the surface occupied by the dural displayed on an axial plane MRI scan (Fig. 2) and measured using the Picture Archiving and Communication System software (Sectra AB Company).

### 2.3 Clinical outcomes

Given the observed improvement in health status among achondroplastic patients following lumbar decompression surgery, follow-up assessments were sent to those maintaining conservative treatment. The investigation included the Oswestry Disability Index (ODI) (Fairbank and Pynsent, 2000), EuroQol-5 Dimensions five levels (EQ-5D-5L) (Solberg et al., 2005), and modified Japanese Orthopaedic Association (mJOA) (Benzel et al., 1991) scores. The ODI involved ten items concerning the intensity of pain, lifting, self-care, ability to walk, ability to sit, sexual function, ability to stand, social life, sleep quality, and ability to travel, and the total score ranged between from 0 (asymptomatic) to 100 (fully disabled). The EQ-5D included a descriptive system and an EQ VAS. The descriptive system covered five dimensions—mobility, self-care, usual activities, pain/discomfort, and anxiety/depression—used to calculate the EQ-5D index, ranging from 1 (full health) to −0.59. The mJOA score was introduced to assess the severity of myelopathy ranging from 0 (fully disabled) to 18 (asymptomatic). The radiological threshold was validated by comparing clinical outcomes between achondroplastic patients with mild and severe lumbar stenosis, categorized by cutoff values of the Schizas scale or DSCA.

### 2.4 Surgical treatment

#### 2.4.1 Decision for surgery

Achondroplasts with complaints of neurogenic claudication were considered for surgery. Complaints comprised pain or exhaustion in the legs during walking and/or standing. The severity of complaints was evaluated in judging limitations of daily activities, pain coping, and influence on the quality of life. Patients with bladder and/or bowel dysfunction, and/or progressive neurological deficit with neurogenic deficit in radicular distribution were indicated for emergency lumbar decompression.

#### 2.4.2 Surgical intervention

Patients were operated under general anesthesia in knee-elbow position if possible. If body composure prohibited this position, patients were operated in a prone position with the lumbar spine in as much flexion as could be effectuated. A lumbar midline incision was made after which muscles were detached from the spinous processes. The spinous process of the upper laminae was reduced and with a diamond burr, the lamina and medial part of the facet joint (zygapophysis) was carefully reduced. Thereafter the ligamentum flavum was reduced until bilateral decompression of the dural sac, lateral recess, and nerve roots was accomplished. Postoperative care was according to the usual spinal surgery protocol, comprising starting mobilization the day after surgery and wound drainage for 12–24 h.

### 2.5 Statistical analysis

Mean and standard deviation were calculated for continuous variables and absolute and relative frequency for categorical variables. Interobserver agreement regarding the Schizas scales (four grades: A, B, C, and D) was calculated using weighted kappa values. The degree of stenosis was demonstrated per lumbar disc level using the Schizas scales and DSCA.

Schizas scales were calculated using weighted kappa values (0–0.20 [slight agreement], 0.21–0.40 [fair agreement], 0.41–0.60 [moderate], 0.61–0.80 [Substantial agreement], 0.81–1.00 [Almost perfect agreement]). Spearman correlation was employed to explore the correlation between the Schizas scales and DSCA (0–0.20 [very weak], 0.21–0.40 [weak], 0.41–0.60 [moderate], 0.61–0.80 [strong], 0.81–1.00 [very strong]). The univariate logistic regression was implemented to assess the association between the clinical relevance (“decompression” or “conservative treatment”) between the Schizas scales. The validity was further estimated by Receiver Operator Characteristics (ROC) curves, cut-off values, the area under the curve (AUC), sensitivity, and specificity with 95 % confidence intervals (CIs). In the same manner, univariate logistic regression combined with ROC validation was performed to investigate the relationship between treatment options and DSCA. All analyses were conducted using R software (v4.1.2; R Core Team, 2023). P values < 0.05 were considered statistically significant.

## 3 Results

A total of 68 patients were included in the study with 21 maintaining conservative treatment and 47 switching to the decompression. There were 75 levels decompressed, and all of these levels could be scored on MRI. In the 21 conservatively treated patients, a total of 220 levels were available for evaluation. The mean age of patients was 46.2 ± 15.4 years, and 60 % of patients were female. Post-operative MRIs were available for 37 patients, and six conservatively treated patients accepted second lumbar MRI evaluation.

### 3.1 Schizas scale evaluation

Interobserver agreement was moderate at the T_12_L_1_, L_12_, and L_45_ levels (0.48, 0.46, and 0.54), substantial at the L_23_ and L_34_ levels (0.63 and 0.61), and almost perfect at the L_5_S_1_ level (0.82). When the Schizas scales were dichotomized into AB and CD grades, the kappa values all reached substantial agreement, ranging from 0.62 to 0.79 (Table 1).

**Table 1:** Demographic data and agreement for the Schizas scales.

Parameters	
Age (years)	46.2 ± 15.4
Percentage of male	27 (39.71 %)
kappa values of 4-grade Schizas scales	
kappa (T_12_L_1_)	0.48
kappa (L_12_)	0.46
kappa (L_23_)	0.63
kappa (L_34_)	0.61
kappa (L_45_)	0.54
kappa (L_5_S_1_)	0.82
kappa values of 2-grade Schizas scales	
kappa (T_12_L_1_)	0.62
kappa (L_12_)	0.63
kappa (L_23_)	0.69
kappa (L_34_)	0.68
kappa (L_45_)	0.63
kappa (L_5_S_1_)	0.79

**Fig. 3:**
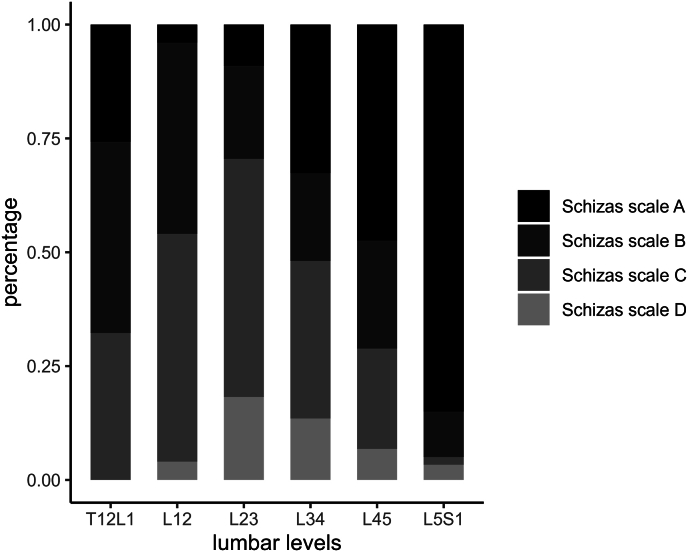
Graphical representation showing the percentage of Schizas scale across the lumbar levels. The proportion of Schizas grades C and D progressively rises from the T_12_L_1_ level to the L_23_ level, then gradually decreases as it moves toward the L_5_S_1_ level.

The highest Schizas grades (narrowest levels) were observed at L_12_ and L_23_ levels. Only two levels were assessed as grade A at the L_12_ level, and four at the L_23_ level (Table 2). Dichotomizing the Schizas scale results into AB and CD revealed that the percentage of grades C and D gradually increased from the T_12_L_1_ level (10/31 = 32 %) to the L_23_ level (31/44 = 70 %) and gradually decreased again in the direction of the L_5_S_1_ level (3/60 = 5 %; Fig. 3).

**Table 2:** Degree of lumbar spine stenosis measured by Schizas scales and dural sac cross-sectional area.

	T_12_L_1_ (n = 31)	L_12_ (n = 50)	L_23_ (n = 44)	L_34_ (n = 52)	L_45_ (n = 58)	L_5_S_1_ (n = 60)
Schizas scales						
A	8	2	4	17	27	51
B	13	21	9	10	14	6
C	10	25	23	18	13	1
D	0	2	8	7	4	2
DSCA (mm^2^)	102.2 ± 44.0	80.2 ± 34.2	67.3 ± 37.7	79.1 ± 47.9	74.3 ± 31.4	102.4 ± 38.1

DSCA, dural sac cross-sectional area.

### 3.2 Evaluation of DSCA

**Fig. 4:**
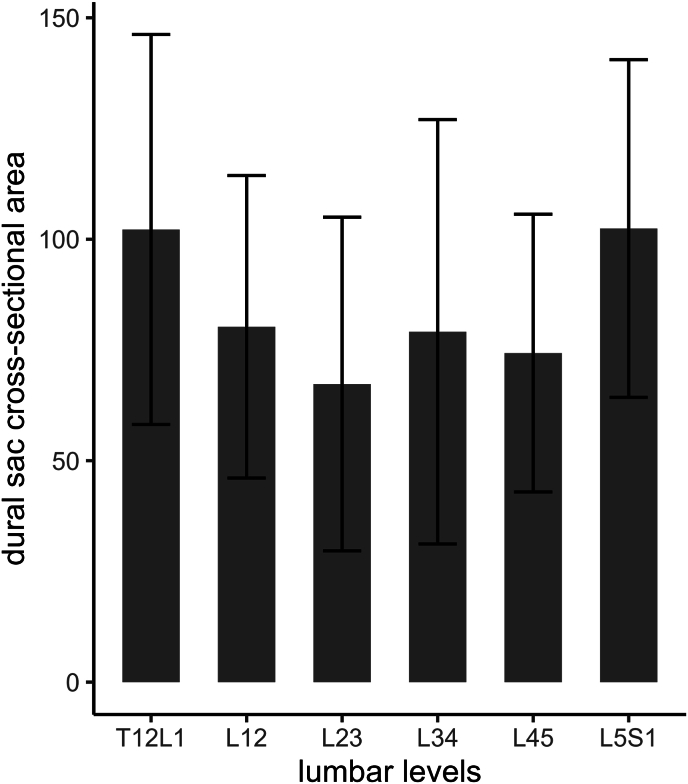
Changes in the dural sac cross-sectional area (DSCA) across lumbar levels. The DSCA decreases from the T_12_L_1_ level toward the L_23_ level, then gradually increases as it approaches the L_5_S_1_ level.

**Fig. 5:**
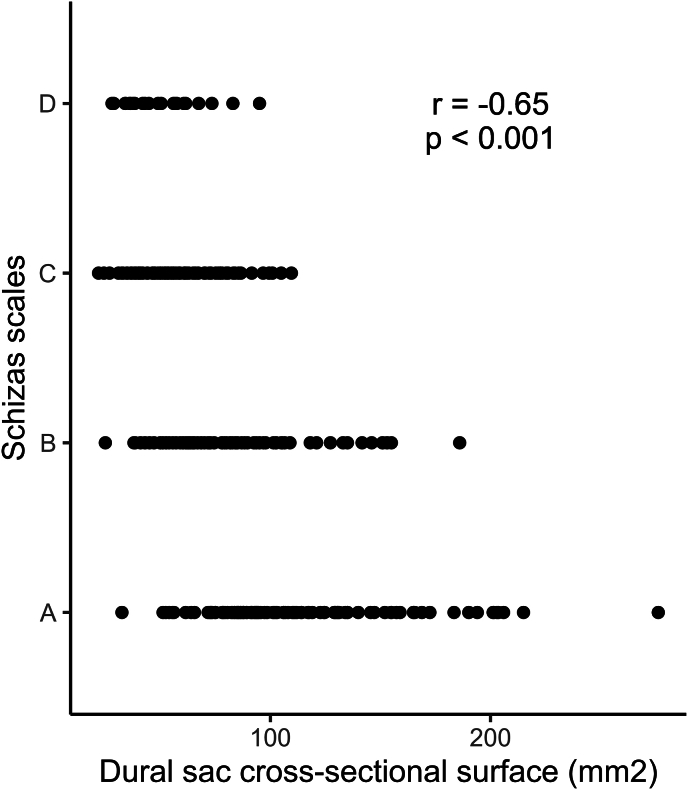
Graphical representation showing the correlation between the dural sac cross-sectional area and Schizas scales. A significant negative association is demonstrated (r = −0.65, p < 0.001).

Likewise, DSCA declined from the T_12_L_1_ level (102.2 ± 44.0 mm^2^) in the direction of the L_23_ lumbar level (67.3 ± 37.7 mm^2^), and gradually increased again downwards to the L_5_S_1_ level (102.4 ± 38.1 mm^2^; Table 2 and Fig. 4). Logically, a significant negative association was identified between the Schizas scales and the DSCA (r = −0.65, p < 0.001, Fig. 5).

### 3.3 Association between the therapeutical approaches and degree of lumbar spinal

The lumbar levels subjected to surgery demonstrated higher Schizas grades (p < 0.001). The majority of surgically decompressed levels were scored with a C (49.3 %), and the majority of conservatively treated levels were scored with an A (44.6 %; Table 3). Likewise, the operated lumbar levels had a smaller DSCA (62.5 ± 34.3 mm^2^ vs 91.0 ± 40.2 mm^2^, p < 0.001, Table 3).

**Table 3:** Comparison of degree of stenosis at the disc levels between accepting decompression and conservative treatment.

	Decompression (75 disc levels)	Conservative treatment (220 disc levels)	P
Schizas scales			<0.001^a^
A	11 (14.7 %)	98 (44.6 %)	
B	17 (22.7 %)	56 (25.5 %)	
C	37 (49.3 %)	53 (24.1 %)	
D	10 (13.3 %)	13 (5.9 %)	
DSCA (mm^2^)	62.5 ± 34.3	91.0 ± 40.2	<0.001^a^

DSCA, dural sac cross-sectional area.

a Statistical significance, p < 0.05.

**Fig. 6:**
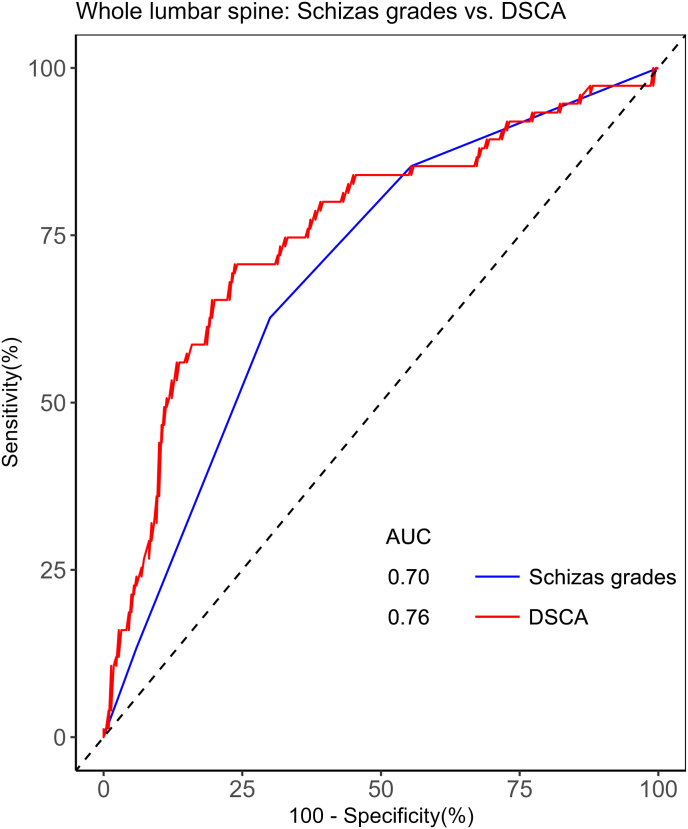
Receiver operating characteristic (ROC) curves for Schizas scale and dural sac cross-sectional area. The ROC curve for Schizas scales (blue line) shows an area under the curve (AUC) of 0.70, and the ROC curve for the dural sac cross-sectional area (red line) presents an AUC of 0.76.

In determining the cutoff values for predicting the appropriate therapy option (decompression or prolonged conservative therapy), univariate binary logistic regression combined with ROC analysis revealed a significant association of Schizas scales with treatment decision (OR 2.10; 95 % CI: 1.57–2.80; p < 0.001). The optimal cutoff value of Schizas scales to predict decompression was grade ≥ C, with 62.67 % sensitivity and 70.00 % specificity (AUC, 0.70; 95 % CI, 0.63–0.76). Similarly, the DSCA presented a significant correlation with the treatment decision (OR 0.97; 95 % CI, 0.96–0.98; p < 0.001). The cutoff value of the DSCA to predict treatment was 62 mm^2^, with 70.67 % sensitivity and 76.36 % specificity (AUC 0.76; 95 % CI, 0.69–0.82, Fig. 6).

One-third of the surgically treated levels (28/75 = 37.3 %) were less stenotic than the Schizas scale threshold (Schizas A or B) and 66 of 220 conservatively treated levels (30 %) demonstrated high Schizas scales (Table 3). Likewise, 22/75 = 29.3 % levels were treated surgically although their DSCA was higher than 62 mm^2^, and 52 of 220 (23.64 %) levels were treated non-surgically, although their DSCA was below the threshold.

### 3.4 Validation of radiological thresholds

To validate the cutoff values of the Schizas scale and DSCA, achondroplastic patients undergoing conservative treatment were categorized based on the severity of lumbar stenosis as either mild or severe. Clinical outcomes were then compared between these groups. Fourteen out of 21 patients receiving conservative treatment responded to the follow-up survey. Among them, 5 patients had the most stenotic level rated as Schizas scale C or D, and 9 patients had the most stenotic level rated as Schizas scale A or B. Additionally, 5 patients had a stenotic level smaller than 62 mm^2^, while 9 had a stenotic level larger than 62 mm^2^. Patients with Schizas scales C or D showed less favorable outcomes, with higher ODI scores (17.6 ± 5.3 vs. 9.4 ± 8.8, p = 0.08), lower mJOA scores (10.3 ± 0.6 vs. 14.6 ± 3.2, p = 0.05), and lower EQ VAS scores (53.0 ± 20.2 vs. 76.7 ± 17.7, p = 0.06). Similarly, patients with DSCA smaller than 62 mm^2^ also exhibited higher ODI scores (16.8 ± 4.5 vs. 9.9 ± 9.4, p = 0.09) and lower mJOA scores (12.7 ± 3.8 vs. 15.0 ± 1.8, p = 0.18, Table 4).

**Table 4:** Comparison of Clinical outcomes between mild and severe stenosis based on the threshold of Schizas scale C and DSCA of 62 mm^2^.

	Schizas scales A or B (n = 9)	Schizas scales C or D (n = 5)	P	DSCA≥62 mm^2^ (n = 9)	DSCA <62 mm^2^ (n = 5)	p
ODI	9.4 ± 8.8	17.6 ± 5.3	0.08	9.9 ± 9.4	16.8 ± 4.5	0.09
mJOA	14.6 ± 3.2	10.3 ± 0.6	0.05	15.0 ± 1.8	12.7 ± 3.8	0.18
EQ-5D index	0.737 ± 0.230	0.619 ± 0.356	0.46	0.646 ± 0.334	0.781 ± 0.076	0.40
EQ VAS	76.7 ± 17.7	53.0 ± 20.2	0.06	69.4 ± 23.8	66.0 ± 18.5	0.79

DSCA, dural sac cross-sectional area; ODI, Oswestry Disability Index; mJOA, Modified Japanese Orthopaedic Association score; EQ-5D, EuroQol 5 Dimensions; VAS, Visual Analogue Scale.

## 4 Discussion

Stenosis in achondroplasia mainly affects the upper lumbar spine, especially the L_23_ level. Previous studies confirmed the area as the most common site for stenosis in achondroplasia (Huet et al., 2020; Calandrelli et al., 2022). Conversely, non-achondroplasts commonly exhibit abnormality in the lower lumbar spine, particularly at the L_45_ level (Kwon et al., 2022). The difference may stem from the persistent thoracolumbar kyphosis in achondroplasts, which alters the shape of the upper lumbar spinal canal.

In applying the Schizas scale to the lumbar spine of achondroplasts, the classification proved to be a more reliable tool than Schizas originally reported in non-achondroplasts. The interobserver agreement in the current evaluation in Ach patients was higher than 0.44 (reported by Schizas) for most lumbar levels, and the correlation with DSCA was found to be significant in contrast to Schizas'results (Schizas et al., 2010). Even still, Schizas' classification is commonly used in grading lumbar stenosis, although the radiograph-clinical correlation was repeatedly reported to be weak when associating the Schizas grades or DSCA with questionnaires, such as the Oswestry Disability Index for non-achondroplasts with lumbar spinal stenosis (Aaen et al., 2022; Weber et al., 2016; Moojen et al., 2018).

DSCA directly measures the degree of stenosis, while Schizas grading classifies the stenosis based on the morphological relationship between nerve roots and cerebrospinal fluid. The present study reported a significant negative association between both parameters. Schizas et al. also reported a similar negative correlation in non-achondroplasts and suggested that the Schizas scale might be a more appropriate grading for LSS than DSCA measurements due to its consideration of neural tissue impingement (Schizas et al., 2010).

The thresholds for surgery in LSS that were estimated in the current paper are in agreement with data that have been published before. Bhalla et al. reported that non-achondroplasts in the United States and Norway have similar radiograph thresholds (Schizas C and D) for surgery. The DSCA less than 75 mm^2^ was linked to an increased risk of exhaustion of conservative treatment in non-achondroplasts (Bhalla et al., 2018). The cut-off value of 62 mm^2^ in achondroplasts reported in this study is logically smaller, due to the deviating anatomy of the achondroplast spine. Having a threshold for surgery would be very practical. However, in evaluating the number of levels that were operated with a deviating score, one-third of surgical levels were below the threshold and one-third of conservative levels were above the threshold. This may partially be explained by the relative position of the level deviating from threshold-prediction. For patients with achondroplasia and multilevel LSS, the surgical strategy prioritizes decompression of the most severely stenotic level to minimize surgical invasiveness and preserve spinal stability. Further intervention is considered only if symptoms persist postoperatively. Otherwise, if a patient has severe complaints of neurogenic claudication and the MRI demonstrates only mild stenosis, it is imaginable that this level is decompressed anyway. It would be interesting to evaluate this clinical outcome of this patient group. Future research should address the issue with the current knowledge, the threshold should be regarded as a guiding decision tool, but not as a gold standard.

To validate the cutoff values, a follow-up investigation was conducted. A trend toward significance was observed in ODI and mJOA scores, suggesting that achondroplastic patients with Schizas scale C or D and a DSCA <62 mm^2^ tend to have poorer clinical outcomes. The ODI score, widely used as a primary outcome in LSS research, reflects daily functioning and is sensitive to health changes, particularly in the context of lumbar dysplasia (Fairbank and Pynsent, 2000).

However, the ODI as a single evaluation tool may not be sufficient. Individuals with achondroplasia are accustomed to coping with limitations. They are often creative in finding solutions to address daily challenges. Therefore, an ODI score – which primarily measures functionality – is not always the most appropriate instrument for assessing well-being. However, as no alternative scale is available, the most informative approach is to interpret the clinical scores in conjunction. Consequently, a quality of life score is at least equally important, and as shown in these scores (Table 4), patients with a higher Schizas grade also report a correspondingly lower quality of life. The mJOA score complements ODI by focusing on neurological impairment due to spinal compression, applicable in both cervical and lumbar stenosis (Benzel et al., 1991). The observed deterioration in all studied clinical outcomes among patients with severe stenosis supports the proposed cutoff values for Schizas scale and DSCA. These thresholds may streamline MRI-based assessment of lumbar stenosis in achondroplastic patients, especially for clinicians without subspecialty training.

In the selection of surgical technique, decompression alone is commonly preferred. Thoracolumbar kyphosis is persistent in patients with achondroplasia. However, spinal stability may still be preserved in these patients, as the TLK is congenital in nature, potentially allowing a certain degree of adaptation to a forward-bending posture. Furthermore, the anatomical constraints of small pedicles and shortened interpedicular distance make fusion technically challenging and may increase the risk of complications.

Despite the strengths of the current study, several limitations should be acknowledged. We acknowledge the lack of preoperative clinical data for surgical patients as a limitation of this study. However, as patient follow-up continues, individuals initially managed non-operatively but later undergoing surgery contribute their earlier questionnaire data as valid baseline measures for surgical outcome evaluation. Secondly, The evaluation of Schizas scale and DSCA of the lumbar spinal canal in the current article focuses on achondroplasts with complaints of neurogenic claudication. Asymptomatic patients or patients with mild symptoms were not enrolled in the study. Thus, the application of the threshold reported by the present study should be limited to symptomatic patients. Thirdly, the axial MR images of several patients did not picture all lumbar levels. The lack of scoring of these (presumably non stenotic) levels undermined the validity of the study. It should be noted though that this is not an argument to scan all levels in the axial plane; tailored to the target radiological examinations should take precedence over the requirement of academic data collection. Furthermore, the issue that remains to be discussed is how to handle the situation when the assessment of Schizas scales and DSCA is conflicting. To address these limitations, we continue to evaluate MRI findings in Ach patients visiting our clinic, to apply the currently presented cut-off values and assess clinical outcome.

## 5 Conclusion

Both Schizas morphological scales and DSCA confirmed that the upper lumbar spine was more frequently affected in adult achondroplasts with LSS. The assessment of cutoff values in Schizas scales and DSCA of lumbar levels in Ach individuals were demonstrated to be correlated to clinically relevant neurogenic claudication requiring surgical decompression. Although the threshold is not strict, it can be regarded as a helpful tool for reviewers of MRIs to diagnose spinal stenosis in achondroplasia patients.

## Funding

This article was funded by 10.13039/501100004543China Scholarship Council (202208110039).

## Conflict of interest-disclosure

The authors declare no conflicts of interest in the preparation of this manuscript.
